# Impaired awareness of hypoglycemia: can structured education address this persistent challenge?

**DOI:** 10.3389/fendo.2026.1722045

**Published:** 2026-03-12

**Authors:** Yuanyuan Chen, Xiaowen He, Yanmin Shan, Meijuan Lan

**Affiliations:** Nursing Department, the Second Affiliated Hospital of Zhejiang University School of Medicine, Hangzhou, China

**Keywords:** challenging issue, hypoglycemia unawareness, impaired awareness of hypoglycemia, new approach, structured education

## Abstract

Impaired Awareness of Hypoglycemia (IAH) describes a condition in insulin-treated diabetic patients characterized by a diminished ability to perceive the onset of acute hypoglycemia. With an estimated prevalence ranging from 23.2% to 26.3%, IAH is associated with a significantly elevated risk of severe hypoglycemia (SH) and is increasingly linked to emotional disturbances. As a major challenge in diabetes management, the primary interventions for IAH include structured education and diabetes technology. However, the real-world impact of diabetes technology on the frequency of SH and IAH remains contentious. While structured education is fundamental for addressing IAH, its implementation is often inadequate, and program content requires optimization, thereby limiting the effectiveness of current educational approaches. Consequently, there is a persistent need for enhanced treatment strategies. With advancements in intelligent technology, a hybrid model integrating structured education with diabetes technology is emerging as a promising trend. Furthermore, IAH can contribute to cognitive dysfunction, which in turn impairs the ability to perceive and respond to hypoglycemic events. This suggests that incorporating cognitive interventions into structured education may represent a novel therapeutic avenue and potentially yield innovative management solutions.

## 1 Introduction

Patients with diabetes who experience frequent hypoglycemia episodes may develop a reduced perception of these events, a condition known as Impaired Awareness of Hypoglycemia (IAH). This was historically termed “unconscious hypoglycemia” or “unawareness hypoglycemia” (1, 2). IAH is formally defined as “diabetes with insulin-treated individuals have diminished ability to perceive the onset of acute hypoglycemia” (2).

IAH is typically assessed using the Gold score or Clarke score, with a threshold of ≥4 indicating the presence of IAH (3, 4). Additional tools for assessing IAH include the Pedersen-Bjergaard method (5) and its modified version (6), the Hypo A-Q score (7), and the Minimally Modified Clarke Hypoglycemia Survey (MMCHS) (8). The Gold score, along with both the original and modified Pedersen-Bjergaard method, consists of a single question, making them suitable for rapid screening (3, 5, 6). Among these, the modified Pedersen-Bjergaard method enables the identification of patients with an intermediate level of awareness, which may lead to finer risk stratification (6). The MMCHS is adapted from the Clarke score, which allows for differentiation between “intrinsic awareness recovery” and “reduction of events attributable to monitoring techniques such as continuous glucose monitoring (CGM)” (4, 8). The Hypo A-Q is a comprehensive and highly sensitive instrument; however, its optimal cut-off value remains to be determined (7). A summary of the key features of these instruments is presented in **Table 1**.

**Table 1:** The information of the scoring methods to assess IAH.

Scoring methods	Number of items	Interpretation of results	Applications
Gold score	1- item	Two categories: Normal awareness (1–3 points)/impaired awareness (≥4 points)	rapid screening, the specificity may be low
Clarke score	8-items	Two categories: Normal awareness (0–3 points)/impaired awareness (≥4 points)	widely used to distinguish states of consciousness from experiences of serious events
Minimally Modified Clarke Hypoglycemia Survey (MMCHS)	8-item	Two categories: Normal awareness (0–3 points)/impaired awareness (≥4 points), A “yes” answer to the question “severe hypoglycemic experience” (question 3 or 4) also allows the diagnosis to be made directly	widely used to distinguish states of consciousness from experiences of serious events. Score improvement due to “intrinsic awareness recovery” and “reduction of events due to monitoring techniques such as CGM” can be distinguished
Pedersen-Bjergaard method	1-item	Three categories: Normal awareness, impaired awareness, loss of awareness	rapid screening, the specificity may be low
Modified Pedersen-Bjergaard method	1-item	Three categories: Normal awareness, intermediate awareness, loss of awareness	Identification of patients in intermediate status may facilitate finer risk stratification
Hypo A-Q score	5-items	The scores ranged from 0 to 20. A higher score represents a greater degree of IAH	The assessment is comprehensive and highly sensitivity, but the cut-off value needs to be determined

## 2 Is IAH a serious health issue?

### 2.1 Epidemiology

The prevalence of IAH differs slightly between type 1 diabetes mellitus (T1DM) and type 2 diabetes mellitus (T2DM), partly due to the differences in residual islet function. T1DM patients are more prone to hypoglycemia, a primary risk factor for IAH (9). A meta-analysis encompassing 21 countries and regions reported IAH prevalences of 23.2% (95% CI: 18.4%–29.3%) using the Clarke score, 26.2% (95% CI: 22.9%–29.9%) using the Gold score, and 58.5% (95% CI: 53.0%–64.6%) according to the Pedersen-Bjergaard method (10). Given the wider recognition of the Gold and Clarke scores, the probable prevalence of IAH is estimated to be between 23.2% and 26.3%.

Another meta-analysis reported a pooled IAH prevalence of 22% (95%CI:14–29%) in patients with T2DM (11). However, prevalence rates vary considerably across regions. For instance, the Dutch Diabetes Pearl Cohort reported IAH in 9.7% of insulin-treated T2DM patients (12), while other studies found prevalences of 28.2% (Clarke-TW) among insulin users (13). 17.01% (13.27%-20.75%) in Edinburgh (14), and as high as 52.1% in Al-Qassim, Kingdom of Saudi Arabia (15). In contrast, European populations generally report lower IAH prevalence (10). This disparity may be attributed to regional variations in T2DM prevalence and more effective T2DM management in developed European countries compared to developing regions (10, 16). Additionally, diabetes duration and insulin use significantly influence these results (11).

IAH is estimated to affect approximately 25% of people with T1DM (17), though significant regional variations exist. The PR-IAH study in Japan reported a prevalence of 11.6% (95% CI: 7.8-16.4%) (18), whereas a study in Saudi Arabia found a significantly higher rate of 62.8% (19). According to the Norwegian childhood diabetes registry, the overall IAH prevalence is 22% (20). Notably, a recent study indicates a declining trend in IAH prevalence among T1DM patients (21), with rates of 32.5% in 2006, 32.3% in 2010, 30.1% in 2016, and 16.0% in 2020, representing an approximately 50% decrease (21). A potential explanation is the increased adoption of glucose sensor technology, used by 85% of T1DM patients, which has been shown to reduce hypoglycemia incidence, particularly SH (21, 22). As the Clarke score heavily relies on a history of SH, the decline in IAH prevalence may partly reflect fewer SH events. Therefore, the trend does not necessarily indicate an improved intrinsic ability to recognize hypoglycemia in T1DM.

### 2.2 Complication risk

IAH is associated with a six-fold increased risk of severe hypoglycemia (SH) (17), with some studies reporting a ten-fold increase (23). A cross-sectional survey further revealed that the proportions of T1DM patients reporting one or more, and two or more, episodes of SH were significantly higher among those with IAH (31.3% and 21.4%, respectively) compared to those without IAH (14.9% and 8.0%) (24). IAH is a well-established major risk factor for SH, as the diminished awareness and the physiological responses to hypoglycemia increase the likelihood of subsequent SH events (25, 26). Frequent hypoglycemia contributes to IAH development, creating a vicious cycle where IAH further impairs hypoglycemia recognition, leading to severe Hypoglycemia and, in extreme cases, death.

Growing evidence also links IAH to emotional disturbances (27). Studies indicate that emotional disorders, including depression and anxiety, are associated with a higher likelihood of IAH. Both conditions were independently associated with increased odds of IAH, with an odds ratio of 3.64 (95% CI: 2.19-6.04) for depression and 2.46 (95% CI: 1.46- 4.14) for anxiety, respectively, and a dose-response relationship has been observed (28). Among T1DM patients using CGM, diminished awareness of hypoglycemia is independently associated with higher anxiety and depression symptoms (29). Emotional disorders can adversely affect diabetes self-care behavior and motivation, impair the ability to distinguish anxiety from hypoglycemia symptoms, delay hypoglycemia treatment, and consequently elevate IAH risk (30). Conversely, recurrent hypoglycemia, or IAH, may also exacerbate emotional issues. The release of central nervous system neurotransmitters related to both hypoglycemia and emotional regulation is a plausible mechanism (31). Fear of hypoglycemia (FoH) is another prevalent emotional response in T1DM. While one might assume that experiencing hypoglycemic symptoms induces FoH, studies show that the FoH score is significantly higher (0.64, 95% CI: 0.45–0.83) among T1DM patients with IAH compared to those with normal awareness (32). Similar findings have been observed in pregnant women with T1DM (33). This counterintuitive result may be because IAH patients experience more frequent SH, which subsequently leads to higher FoH (34). Furthermore, higher levels of anxiety and depression are also correlated with increased FoH (35). In summary, IAH directly or indirectly influences anxiety, depression, and FoH.

## 3 Whether structured education can improve IAH?

Structured education programs for IAH, including comprehensive self-management and IAH treatment programs, are acknowledged for their potential to enhance awareness of hypoglycemia. Although comprehensive self-management programs are not specifically designed for IAH, they can positively influence IAH by reducing hypoglycemia. These include Dose Adjustment For Normal Eating (DAFNE) (36), DAFNEplus (37), the German Diabetes Treatment and Training Program (DTTP) (38), and derivative courses such as the Tayside insulin management course (39), Bournemouth Type 1 Intensive Education (BERTIE) (40,) and a Self-management-oriented Education Programme (PRIMAS) (41). IAH treatment programs are developed to address IAH. These include Blood Glucose Awareness Training (BGAT) (42), Hypoglycemia Anticipation, Awareness and Treatment Training (HAATT) (43), HypoAware (44), Hypoglycemia Treatment Programme (HyPOS) (45), ‘My Hypo Compass’ short psychoeducational intervention (46), and Hypoglycaemia Persisting Despite Optimized Self-care (HARPdoc) (37). Structured education can ameliorate IAH by preventing hypoglycemia and improving hypoglycemia recognition skills (36–46). While beneficial for IAH patients, the extent of its effectiveness remains uncertain.

### 3.1 Comprehensive self-management program as a foundation for IAH

A comprehensive self-management program is essential for T1DM. However, few patients daily adjust their insulin regimen or achieve ideal glycemic control (47). Dietary restrictions imposed to accommodate fixed insulin regimens can negatively impact patients’ quality of life (47). Consequently, a comprehensive self-management program, such as the five-day structured education program, like DAFNE or DTTP, is widely adopted to increase dietary freedom and improve quality of life (36–39). DTTP is an inpatient, Monday-to-Friday, group teaching program that serves as a foundational and routinely delivered diabetes education course (38). DAFNE, a curriculum delivered within the UK National Health Service (NHS), employs a progressive, modular-based structure to enhance self-management across a range of medical and social situations (48). Its core modules include: what is diabetes; food and diabetes; insulin management; management of hypoglycemia; sick day rules (48). Although DAFNE has been shown to improve both glycemic control and quality of life, more than half of its graduates continue to struggle with maintaining glucose levels (49). To solve this limitation, the DAFNE plus intervention is developed as an enhancement of the original DAFNE program, incorporating behavioral change techniques, technology, and longer-term structured support from healthcare professionals (50). In contrast, other courses, such as the Tayside insulin management course (39), the BERTIE (40), and the PRIMAS (41), face regional and linguistic limitations. Notably, the PRIMASA program emphasizes addressing emotional challenges, which is critical given the chronic, lifelong nature of diabetes and its associated emotional distress and psychological burden (51). Therefore, greater attention to psychological aspects, beyond mere blood glucose control, is imperative.

As a form of structured education, comprehensive self-management programs aim not only to control blood glucose but also to prevent IAH. These programs teach patients to adjust insulin doses based on food intake rather than adapting meal timing and content to fixed insulin doses (52). In a DTTP study, sensitivity to low blood glucose (BG) levels (49.1 ± 4.2 vs. 54.9 ± 4.9, *P* = 0.12) did not change significantly. However, patients with a history of repeated SH demonstrated improved accuracy in BG estimation (24.8 ± 6.2 VS 36.9 ± 8.3, *P* = 0.04), whereas those without such a history did not show significant improvement (48.5 ± 3.9 VS 46.9 ± 4.6, *P* = 0.5). In DAFNE research, 43% of participants with IAH at enrollment reported restored ability to detect hypoglycemia at BG levels >3 mmol/L, and these patients experienced a significant reduction in SH episodes (36). This indicates that comprehensive self-management programs can reduce SH rates. However, their effect on IAH is limited, likely because these programs were primarily designed for overall BG management of T1DM rather than specifically for IAH.

Overall, comprehensive self-management programs share a common goal: to help patients achieve a more flexible lifestyle, provided that it does not increase the risk of hypoglycemia. Then, these programs are not one-off interventions. Except for DTTP, which follows a classic 5-day inpatient model, most are delivered as one day per week over four or five weeks. This design allows participants sufficient time to apply the skills learned in class (e.g., carbohydrate counting, dose adjustment) to their daily life, and to bring questions and experiences back for discussion in subsequent sessions. By integrating theory with practice, this approach enhances learning outcomes. However, strong peer support is essential to maintain engagement and achieve a low dropout rate (53). Finally, comprehensive self-management programs, particularly DWNE, should be considered a compulsory component of diabetes self-management education. Even if they do not directly target IAH, they serve as an essential foundation for IAH intervention.

### 3.2 IAH treatment programs and their potential to improve hypoglycemia awareness

IAH plays a major role in the etiology of hypoglycemic problems, with patients exhibiting a markedly increased risk for SH (23–26). Given that comprehensive self-management programs alone may be insufficient, specialized IAH treatment programs have been developed. BGAT is a classic course designed to enhance patients’ awareness of BG fluctuations, particularly for T1DM patients with hypoglycemia or hyperglycemia unawareness (42). BGAT has three versions: the original BGAT-1 with six chapters focusing on internal cues (42); BGAT-2 with eight chapters emphasizing both internal and external cues (54); and BGAT-3, an updated version that incorporates newer insulin formulations and addresses long-term maintenance in Chapter 8 (46). The efficacy of BGAT is well documented (42, 54, 55), demonstrating benefits that include improved BG estimation accuracy and reduced SH frequency. While BGAT aims to assist T1DM patients in better identifying the symptoms of hypoglycemia and hyperglycemia (42), it has been noted that there is only a negligible benefit in improving the detection of hypoglycemia (56). The HyPOS is another notable course specifically targeting the prevention of low BG levels and educating patients about IAH causes (45). Compared to BGAT, HyPOS showed a great improvement in the hypoglycemia awareness questionnaire (HAQ) score (difference 0.7 [*95% CI* 0.1–1.2], *p* = 0.024), and the visual analogue scale (VAS) score (difference 0.8 [*95% CI* 0.2–1.2], *p* = 0.015) (45). Although both BGAT and HyPOS demonstrate efficacy, their uptake in routine diabetes care remains low, likely due to the demanding nature of face-to-face education, which appears too demanding for widespread implementation in diabetes management (44). To address this, HypoAware was developed as an adaptation of BGAT, incorporating online modules to enhance accessibility awareness training (57). HypoAware significantly reduced the odds of impaired awareness (*OR* = 0.38, *95% CI* 0.15–0.95, *P* = 0.038), though it did not significantly decrease IAH frequency(81% VS 68%, *P* = 0.227) (44). Nonetheless, adherence to the HypoAware program was good (44, 58). As digitalization accelerates, integrating online and offline education has become essential. Therefore, developing multi-channel structured educational courses, while ensuring efficacy, can reach a broader population of IAH patients.

HARPdoc is an innovative psychoeducational intervention program that integrates psychological and cognitive approaches (37). This program is specifically designed for patients with IAH who have a history of recurrent SH, despite having attended structured education (such as DAFNE courses) and utilized advanced technologies, including continuous glucose monitoring or insulin pumps (59). Although HARPdoc is not superior to BGMT in reducing SH, it significantly lowered diabetes-related distress, depression, and anxiety scores (60, 61). Given that patients with IAH often present with psychological comorbidities such as anxiety, depression, and FoH (30, 32), targeted psychological-cognitive interventions are of importance. Other structured education programs include HAATT (43), DAFNE-Hypoglycemia Awareness Restoration Training (DAFNE-HART44, and ‘My Hypo Compass’ short psychoeducational intervention (46). However, many of these programs have been evaluated primarily in small-sample exploratory studies with regional limitations, requiring further verification.

In summary, IAH treatment programs share a core philosophy: to improve patients’ perception of and response to glycemic fluctuations, with BGAT serving as the foundational model. Then, the focus has evolved to address deeper psychological and cognitive issues, as exemplified by HARPdoc, which specifically targets unhelpful health beliefs underlying persistent IAH. Last, the delivery format of these interventions has diversified, evolving from traditional face-to-face group courses (e.g., HyPOS) to online modules (e.g., HypoAware) and brief psychoeducational tools (e.g., My Hypo Compass). This evolution reflects an ongoing effort to enhance accessibility, scalability, and convenience for broader patient populations. Therefore, an ideal structured education program for IAH should encompass core educational content, adopt diverse delivery formats that integrate both traditional models and intelligent technologies, and place sufficient emphasis on psychological and cognitive dimensions.

## 4 Concluding remarks: what shall we do?

### 4.1 The hybrid model: integrating structured education and diabetes technology

With the growing burden of diabetes, the importance of addressing IAH is increasingly emphasized. Exploring more effective interventions to enhance hypoglycemia recognition remains crucial. Alongside structured education, diabetes technology- including CGM, insulin pump, and automated insulin delivery (AID) systems like hybrid closed-loop(HCL) systems- constitutes another significant intervention for IAH and has become integral to diabetes management (62). Research involving T1DM patients has demonstrated that CGM use can lead to sustained improvements over two years (63). In the real-world clinical settings, emerging evidence suggests that an advanced HCL system may improve hypoglycemia awareness in adults with T1DM, particularly among those with IAH (Clarke’s score from 3.6 ± 0.8 at T0 to 1.9 ± 1.6 at T6, P < 0.001) (64). Another study observed a lower median Gold score in HCL users compared to non-users [4.0 (IQR:3.0-5.5) versus 5.5 (IQR:4.5-6.0), *P* = 0.033], suggesting potential benefits of an HCL system in this population (65). However, the evidence remains inconclusive, and researchers hold divergent views regarding the efficacy of HCL for improving IAH. A retrospective, observational, cross-sectional study found that a substantial proportion of patients with T1DM continue to experience severe hypoglycemic events (SHEs) and IAH, despite the use of advanced diabetes technologies (24). In high-risk adults with severe IAH, six months of HCL use did not increase hypoglycemia and partially restored counter-regulatory hormone responses; however, no significant changes were observed in Clarke or Gold scores (66). These findings suggest that the real-world impact of diabetes technology on IAH remains controversial, highlighting the need for improved future strategies. Notably, diabetes technologies are primarily designed to optimize glycemic control and minimize hypoglycemia, rather than directly enhance hypoglycemia awareness. Therefore, integrating structured education with diabetes technology may be essential to both prevent hypoglycemia and address IAH concurrently. A systematic review and meta-analysis also demonstrated that HCL has the potential to improve hypoglycemia awareness in patients with T1DM and IAH, but the clinical significance of this effect may be limited (67). Therefore, educational interventions remain the cornerstone of IAH management. Accordingly, effective approaches to integrating education and technology warrant further exploration.

### 4.2 A new approach: incorporating cognitive function interventions into structured education?

Diabetic patients with IAH experience an accelerated decline in cognitive function and an increased risk of dementia (68, 69). This decline does not indicate an acute cognitive impairment during hypoglycemic disorders with disturbed consciousness. Rather, it manifests as a form of memory interference, wherein similar hypoglycemia events are encoded as shared representations rather than as distinct, episodic memories (69, 70). Consequently, IAH patients are less able to distinguish hypoglycemia-related cues, hindering appropriate actions to avoid SH (69, 70). Additionally, their planning abilities are often compromised, preventing behavioral modifications to avert hypoglycemia (69, 70). Neurophysiological studies reveal that diminished cerebral blood flow responses in regions such as the thalamus, frontal lobe, and hippocampus in IAH patients (55). This disruption affects pathways related to arousal, decision-making, and reward processing (71), potentially impairing the ability to recognize and manage hypoglycemia effectively (71). Recent studies have identified differences in brain activation regions, suggesting that neuroimaging variations may explain differing behavioral responses to acute hypoglycemia (31, 72). Thus, these studies of neural mechanisms all point to one conclusion that IAH can lead to cognitive dysfunction, which in turn diminishes hypoglycemia perception and processing capabilities (73, 74). Clinical research on the relationship between IAH and cognitive function has revealed that patients with IAH exhibit impairments across multiple cognitive domains, including diminished learning and memory, reduced pattern separation ability, behavioral dysfunction, and slower processing speed and attention task performance (70, 75). However, longitudinal cohort studies tracking cognitive function in patients with IAH over extended periods remain scarce, precluding definitive conclusions regarding the specific nature of the relationship between IAH and cognitive decline. Nonetheless, drawing upon existing evidence from neural mechanisms and cross-sectional clinical research, it is reasonable to hypothesize that recurrent hypoglycemia episodes may lead to progressive cognitive deterioration. This decline could, in turn, impair patients’ ability to recognize key elements of hypoglycemic cues, ultimately contributing to the development or perpetuation of IAH. This intriguing potential pathway suggests that cognitive interventions—particularly those targeting learning, memory, pattern separation, behavioral regulation, processing speed, and attention—may enhance hypoglycemia perception in individuals with IAH (75, 76). Given this hypothesis, long-term monitoring of cognitive function in patients with frequent hypoglycemia is of considerable clinical importance. Such surveillance could enable the timely identification of cognitive decline and inform targeted cognitive interventions. This represents a novel and testable hypothesis that warrants further exploratory clinical research to validate its premises and therapeutic implications.

## 5 Key messages

In summary, comprehensive self-management programs (e.g., DAFNE, DTTP) provide the essential foundation of diabetes care and indirectly improve IAH by reducing hypoglycemia risk, but many patients continue to face glycemic instability.

IAH treatment programs (e.g., BGAT, HARPdoc) directly address IAH; HARPdoc, in particular, significantly reduces diabetes-related distress, depression, and FoH. To enhance the effectiveness of structured education, clinicians should also support families by addressing their psychosocial, behavioral, and practical needs (77).

**Figure 1:**
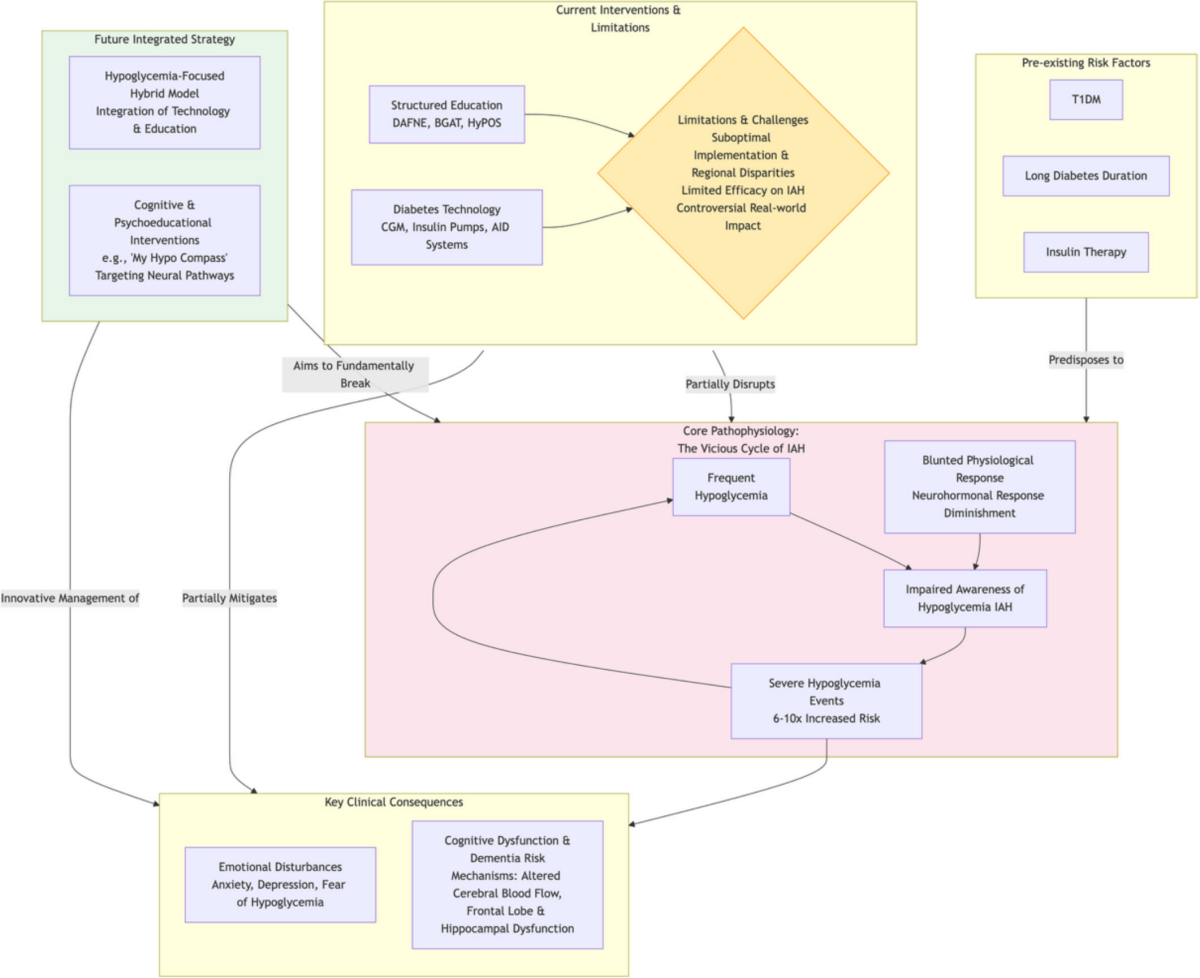
The Pathophysiology, Consequences, and Evolving Management Paradigm of IAH.

Advanced diabetes technologies (e.g., hybrid closed-loop systems) reduce hypoglycemia exposure but do not restore hypoglycemia awareness, highlighting that technology alone is insufficient. Emerging evidence suggests that recurrent SH may impair cognitive functions, such as learning, memory, and pattern separation, potentially perpetuating IAH. This positions cognitive training as a promising novel therapeutic avenue. Future research should prioritize long-term prospective studies monitoring cognitive function in high-risk patients to clarify causality and guide early intervention. Ultimately, an integrated approach combining structured education, psychological-cognitive support, and intelligent technology—delivered flexibly—represents the optimal strategy for IAH management (**Figure 1**).
